# Supplementary material for: Food Insecurity and Child Development: A State-of-the-Art Review
Source: Int J Environ Res Public Health. 2021 Aug 26;18(17):8990. doi: 10.3390/ijerph18178990 (PMC8431639; doi:10.3390/ijerph18178990)
Supplement: Supplementary file 1 [file ijerph-18-08990-s001.zip › Supplementary Table S2.pdf]

Supplementary Table S2

Quality assessment of included studies

Study reference	Country	Scope	Study Period	Study Type	Study/Sampling	NIH Rating
Black (2012)	USA	7 cities	2000-2010	Cross-sectional	Children's HealthWatch	Fair
Cook (2013)	USA	7 cities	1998-2011	Cross-sectional	Children's HealthWatch	Fair
Drennen (2019)	USA	7 cities	2009-2017	Cross-sectional	Children's HealthWatch	Good
Encinger (2020)	USA	2 cities	2014-2015	Cross-sectional	Three child-care centres	Fair
Gee (2018)	USA	National	2010-2011	Longitudinal	ECLS-K	Good
Grineski (2018)	USA	National	2010-2011	Longitudinal	ECLS-K	Good
Hobbs (2018)	USA	National	1998-2005	Cross-sectional	Fourth wave of Fragile Families and Child Wellbeing Study	Good
Howard (2011)	USA	National	1999-2003	Longitudinal	ECLS-K	Fair
Huang (2016)	USA	National	1998-2004	Longitudinal	ECLS-K	Good
Huang (2018)	USA	National	2005	Cross-sectional	48month wave of ECLS-B	Fair
Jackson (2018)	USA	National	1998-2010	Longitudinal	Fragile Families and Child Wellbeing Study	Good
Johnson (2017)	USA	National	2001-2007	Longitudinal	ECLS-B	Good
Kimbro (2015)	USA	National	2010-2012	Longitudinal	ECLS-K	Fair
King (2018)	USA	National	2001-2003	Longitudinal	Fragile Families and Child Wellbeing Study	Good
Melchior (2012)	Canada	Quebec	1997-2006	Longitudinal	Quebec Longitudinal Study of Child Development	Fair
Nagata (2018)	USA	San Francisco	2006-2007	Cross-sectional	Latina women attending prenatal clinics	Fair
Ramsey (2011)	AUS	Brisbane	2009	Cross-sectional	Postal survey in disadvantaged areas	Fair
