# Supplementary material for: Food Insecurity and Child Development: A State-of-the-Art Review
Source: Int J Environ Res Public Health. 2021 Aug 26;18(17):8990. doi: 10.3390/ijerph18178990 (PMC8431639; doi:10.3390/ijerph18178990)
Supplement: Supplementary file 1 [file ijerph-18-08990-s001.zip › Supplementary Table S1_Syntax.pdf]

Supplementary Table S1: Search and Boolean Terms

Database	Search Terms
Medline	**S1** MH "Child Development" OR (TI ((child* OR infant* OR p#ediatric) N3 (outcome* OR health* OR development*))) OR (AB ((child* OR infant* OR p#ediatric) N3 (outcome* OR health* OR development*)))
Medline	**S2** TI (food N2 (secur* OR insecur* OR availabil* OR unavailabil* OR access* OR expenditure OR afford* OR utili?ation OR literac* OR sufficien* OR insufficien* OR poverty OR barrier* OR desert)) OR AB (food N2 (secur* OR insecur* OR availabil* OR unavailabil* OR access* OR expenditure OR afford* OR utili?ation OR literac* OR sufficien* OR insufficien* OR poverty OR barrier* OR desert))
Medline	**S3** MH "Food Habits+" OR MH "Food Supply" OR MH "Nutritional Status"
Medline	**S4** S1 AND S2 AND S3
PQ Health & Medical Complete; Nursing & Allied Health Source	**S1** MESH.EXACT("Child Development") OR ((child* OR infant* OR p*ediatric) NEAR/3 (outcome* OR health* OR development*))
PQ Health & Medical Complete; Nursing & Allied Health Source	**S2** food NEAR/2 (secur* OR insecur* OR availabil* OR unavailabil* OR access* OR expenditure OR afford* OR utili?ation OR literac* OR sufficien* OR insufficien* OR poverty OR barrier* OR desert)
PQ Health & Medical Complete; Nursing & Allied Health Source	**S3** MESH.EXACT("Food Habits") OR MESH.EXACT("Food Supply") OR MESH.EXACT("Nutritional Status")
PQ Health & Medical Complete; Nursing & Allied Health Source	**S4** S1 AND S2 AND S3
PQ Health & Medical Complete; Nursing & Allied Health Source	**S5** S4 AND la.exact("ENG")
PQ Psych, Soc. Sc., Educ., & Diss. And Theses Global	**S1** Exact("child development") OR (child* OR infant* OR p*ediatric) NEAR/3 (outcome* OR health* OR development*)
PQ Psych, Soc. Sc., Educ., & Diss. And Theses Global	**S2** food NEAR/2 (secur* OR insecur* OR availabil* OR unavailabil* OR access* OR expenditure OR afford* OR utili?ation OR literac* OR sufficien* OR insufficien* OR poverty OR barrier* OR desert)
PQ Psych, Soc. Sc., Educ., & Diss. And Theses Global	**S3** Exact("food" OR "food supply" OR "nutritional status")
PQ Psych, Soc. Sc., Educ., & Diss. And Theses Global	**S4** S1 AND S2 AND S3
PQ Psych, Soc. Sc., Educ., & Diss. And Theses Global	**S5** S4 AND la.exact("ENG")

Database	Search Terms
PsycINFO	**S1** DE "Childhood Development" OR (TI ((child* OR infant* OR p#ediatric) N3 (outcome* OR health* OR development*))) OR (AB ((child* OR infant* OR p#ediatric) N3 (outcome* OR health* OR development*)))
PsycINFO	**S2** TI (food N2 (secur* OR insecur* OR availabil* OR unavailabil* OR access* OR expenditure OR afford* OR utili?ation OR literac* OR sufficien* OR insufficien* OR poverty OR barrier* OR desert)) OR AB (food N2 (secur* OR insecur* OR availabil* OR unavailabil* OR access* OR expenditure OR afford* OR utili?ation OR literac* OR sufficien* OR insufficien* OR poverty OR barrier* OR desert))
PsycINFO	**S3** DE "Food Intake" OR DE "Food Preferences" OR DE "Nutrition" OR DE "Nutritional Deficiencies"
PsycINFO	**S4** S1 AND S2 AND S3
Scopus	**S1** TITLE-ABS-KEY(food W/2 secur*) OR (food W/2 insecur*) OR (food W/2 availabil*) OR (food W/2 unavailabil*) OR (food W/2 access*) OR (food W/2 expenditure) OR (food W/2 afford*) OR (food W/2 utili?ation) OR (food W/2 literac*) OR (food W/2 sufficien*) OR (food W/2 insufficien*) OR (food W/2 poverty) OR (food W/2 barrier*) OR (food W/2 desert)
Scopus	**S2** TITLE-ABS-KEY(child* W/3 outcome*) OR (child* W/3 health*) OR (child* W/3 development*) OR (infant* W/3 outcome*) OR (infant* W/3 health*) OR (infant* W/3 development*) OR (p*ediatric W/3 outcome*) OR (p*ediatric W/3 development*) OR (p*ediatric W/3 health*)
Scopus	**S3** S1 AND S2
Web of Science	**S1** TS=(child NEAR/3 outcome* OR child NEAR/3 health* OR child NEAR/3 development* OR infant NEAR/3 outcome* OR infant NEAR/3 health* OR infant NEAR/3 development* OR p$ediatric NEAR/3 outcome* or p$ediatric NEAR/3 development* OR p$ediatric NEAR/3 health*)
Web of Science	**S2** TS=(food NEAR/2 secur* OR food NEAR/2 insecur* OR food NEAR/2 availabil* OR food NEAR/2 unavailabil* OR food NEAR/2 access* OR food NEAR/2 expenditure OR food NEAR/2 afford* OR food NEAR/2 utili?ation OR food NEAR/2 literac* OR food NEAR/2 sufficien* OR food NEAR/2 insufficien* OR food NEAR/2 poverty OR food NEAR/2 barrier* OR food NEAR/2 desert)
Web of Science	**S3** S1 AND S2
